# The Influence of Target Layout and Clicking Method on Picking Time and Dragging Performance Based on Eye-Control Technique

**DOI:** 10.3389/fpsyg.2020.01618

**Published:** 2020-07-15

**Authors:** Lian Wang, Dan Wang, Yingwei Zhou, Haixiao Liu, Jinshou Shi, Yuting Zhao, Chi Zhang, Jianwei Niu

**Affiliations:** ^1^School of Mechanical Engineering, University of Science and Technology Beijing, Beijing, China; ^2^China Institute of Marine Technology and Economy, Beijing, China

**Keywords:** eye-control technique, target clicking method, target layout, human–computer interaction, picking time, dragging performance

## Abstract

Eye-tracking has been a hot topic in human–computer interaction (HCI). Nevertheless, previous studies usually adopted eye-tracking as information output rather than input. The eye-control technique can achieve convenient and rapid real-time operation through the movement of the eyes and reduce unnecessary manual operations. Because the layout determines the location orientation, organizational complexity, cognitive consistency, and predictive ability of the information display, the interface layout design affects the user’s perception of information intensity, complexity, and logic. Moreover, the method of target clicking by eye-control techniques, which include blink and dwell, also depends on the application and user’s ability. The purpose of this study is to investigate the influence of target layout and target picking method on picking time and dragging performance based on eye-control technique. The results indicate that the target picking method, i.e., blink or dwell, had significant effects on the dragging time and dragging numbers. However, there was no significant effect of target layout on picking time and dragging performance (dragging time and numbers), which may be related to the setting of the experimental conditions (e.g., lighting level and screen resolution). Moreover, the target picking method and the target layout had no significant interaction effect on picking time and dragging performance. The findings are anticipated to provide helpful implications for future eye control technique design.

## Introduction

Eye-tracking technology has spread widely in the last decade, but it is seldom reported that eye-control techniques are used in either academia or industrial applications. Eye control is an advanced technique in human–computer interaction (HCI) research that can achieve convenient and rapid real-time operation through the movement of the eyes and reduce unnecessary manual operations. With the deepening of eye movement sensing and pattern recognition, this technology is most widely used in education- (Wu, 2012), medicine- (Harezlak and Kasprowski, 2018), military- (Muehlethaler and Knecht, 2016), entertainment- (Lin et al., 2004), and psychology-related fields (Renshaw et al., 2003).

There are some different types of eye movement that were identified by previous researchers, some of which keep the fovea on a visual target in the environment (e.g., saccades and smooth pursuits), while others stabilize the eye during head movement (e.g., fixations) (Underwood and Radach, 1998; Duchowski, 2007; Liversedge et al., 2011). Through these different types of eye movements, the eye can complete the aiming and continuous dynamic observation of the object, thus ensuring clear visual input. The eye tracker is a complex and precise psychological instrument. It can measure individual eye movement characteristics and evaluate the validity of effective interfaces by visual cognitive physiological evaluation (Zhao et al., 2018). Eye tracking techniques have been applied to investigate HCIs (Jacob and Karn, 2003; Goldberg and Helfman, 2011).

In graphical user interaction, spatial attributes (such as topology, geometry, spatial relationships, etc.) are mapped to functional attributes (such as causality, hierarchical relationships, associations, etc.). For example, Liu et al. (2016) pointed out that stable covariant structural information can reduce the complexity of the scene while increasing its predictability. Because the layout determines the location orientation, organizational complexity, cognitive consistency, and predictive ability of the information displayed, the interface layout design affects the user’s perception of information intensity, complexity, and logic. In the 1950s, Fitts et al. (1949, 1950) studied the series of eye movements in the pilot landing process to determine an effective method of assessing the importance of the instrument, the difficulty of instrument reading, and the instrument layout design by eye-moving techniques. Eye movements are thought to provide an indication of the amount of cognitive processing display requirements (Raynor and Pollatsek, 1994), and eye tracking can be a tool for the assessment of usability (Renshaw et al., 2003; Rayner, 2009). Pušnik et al. (2016) studied how layout, typeface use, position of titles and/or text, color combination draw attention and affect the recall of presented content by using eye-tracking technology. Nevertheless, previous studies usually adopted eye-tracking as information input rather than eye control as information output. How users interact with the graphic user interface (GUI) and what kinds of GUI parameters influence the interaction performance deserve deep investigation.

Because the layout determines the location orientation, organizational complexity, cognitive consistency, and predictive ability of the information display, the interface layout design affects the user’s perception of information intensity, complexity, and logic. Moreover, the method of target clicking by eye-control techniques, which include blink and dwell, also depends on the application and user’s ability. Thus, we proposed the hypotheses as follows:

H1: The increase of operation repetitions had significant effects on the performance.
H2: The target layout had significant effects on the dragging time and dragging numbers.
H3: The target picking method (blink or dwell) had significant effects on dragging performance.
H4: The target picking method and the target layout had no significant interaction effect on picking time and dragging performance.

This paper aimed to investigate the influence of target layout and target clicking method on picking time and dragging performance based on eye-control technique. Through the study of visual perception and information processing mode, we explored whether the position on the screen and the target clicking method would affect the operation process. The results could provide some suggestions for the design of human-machine interfaces and clicking methods that are more suitable for eye-control systems.

## Materials and Methods

### Experiment Design

We performed a repeated measures experiment to investigate the influence of target layout and target clicking method on picking time and dragging performance based on eye-control technique. The independent variables were the initial position of the target, which had five values (screen center, top left, bottom left, top right, and bottom right), and the method of target picking, which had two values: *blink* (blinking twice as a click) and *dwell* (focusing the eyes on the target for 1 s as a click). The dependent variables were the picking time (the time of picking the target successfully), the dragging time (the time of dragging the target into the specified range and dropping successfully), and the dragging numbers (the number of targets successfully dragged the target into the specified range). The last two variables were considered variable indicators of dragging performance.

### Task Design

**FIGURE 1:**
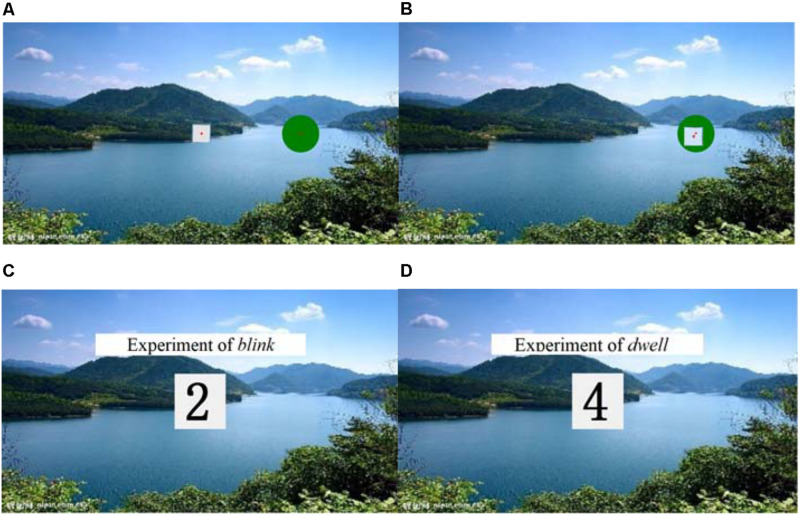
The experimental user interfaces. **(A)** Initialization interface. **(B)** Putting the target square into the circular range. **(C)** Interactive interface of *blink*. **(D)** Interactive interface of *dwell*. Reproduced with permission from Springer Nature.

The target square has five positions: screen center, top left, bottom left, top right, and bottom right. A set of a target square (one of the five positions) and a circular target (around the target square) is randomly displayed on the screen. The interactive interface is shown in Figure 1A. The participants were asked to use eyeball movement to drag the target into the specified circular range and drop the target. The operation of successfully putting the target square into the circular range is shown in Figure 1B. The participants completed the target picking and dropping by two different methods, *blink* (blinking twice as a click) and *dwell* (focus the eyes on the target for 1 s as a click). The interactive interface of *blink* and *dwell* is shown in Figures 1C,D.

### Participants

In the experiment, we recruited 16 participants between 20 and 27 years old, with an average age of 22 years. The participants were university students and working engineers from the Institute of Marine Technology and Economy, China. All participants had not been familiar with the experiment in advance, and all participants were required to maintain normal eyesight without glasses. We randomly select the participant to complete the experiment independently.

### Experimental Facility

We chose the Tobii Eye Tracker 4C for eye movement control which can track the eye and head at the same time. The parameters of the eye tracker are as follows: operating distance: 20–37”/50–95 cm, and image sampling rate: 90 Hz. The working principle of the eye tracker is: the projector projects the pattern into the eye through near infrared rays, the cameras take high-resolution images of the user’s eyes and pattern, the image processing algorithm finds specific details in the user’s eyes and reflection mode, based on this information the eye’s position and gaze point are calculated.

In addition, we chose a 15.6-in display notebook to connect the eye tracker, and the resolution of the notebook is 1920 × 1080. The Tobii Eye Tracker 4C only needs to be fixed under the experimental computer screen, which is a good solution to reduce the experimental error caused by the uncomfortable side effects of long-time experiment.

Clear View data analysis software was used to analyze eye movement data, the interface, and the video of the user’s action.

### Experiment Procedure

**FIGURE 2:**
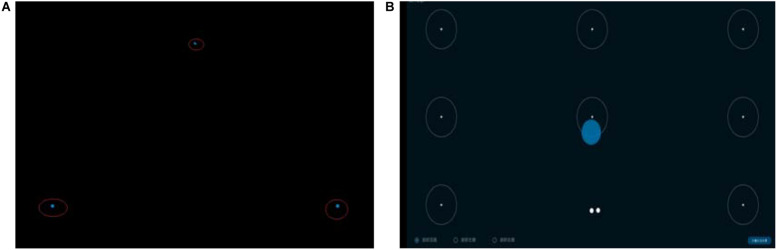
Eye tracker calibration and test interface. **(A)** *Calibration* interface. **(B)** *Test* interface.

The participants were asked to complete a profile questionnaire (demographic information) and an informed consent form first. Then, experiment leader explained the experimental purpose and procedure to the participants. Then the formal experiment started. First, eye movement calibration was conducted. The participants were told to sit upright in the chair with their eyes facing the front, and to try to maintain a stable posture. The experimenter calibrated the eye gaze for the participant. During the calibration process, the participants were reminded that there would be three groups of dots in different corners on the screen. The calibration interface is as shown in Figure 2A. After the calibration, we tested the calibration effect. By looking at the calibration points in the circle, we can determine whether the calibration was accurate enough or not. If the gaze point was in the circle, the calibration was considered good enough; otherwise, we would perform the calibration once again. The test interface is as shown in Figure 2B.

Next, a set of target squares (one of five positions) and a circular target (around the target square) would be randomly displayed on the screen. The participant was asked to use eye movement to drag the target to the specified circular range, and then to drop the target. Participants were required to complete two sets of experiments with two clicks (blink and dwell). After a set of a target square and a circular target was displayed on the screen, the participants fixated on the target square for one second to pick up the target square. Then, he/she needed to drag the target square to the circular range and put it down by fixating for one second. This was a complete operation. Participants could drag and drop multiple times until the target square was successfully placed in the circular range. Three seconds after the completion of one operation, the target would randomly appear in the next position. The operation in five locations is one group of experiments, and the experiment was repeated for four groups, i.e., 20 trials per participant in total for each target clicking method. After the dwell experiment was completed, the target clicking method changed to blink, and the experimental procedure remained the same.

### Data Analysis

We performed a repeated measures analysis of variances (RMANOVA) and categorical regression with optimal scaling (CATREG) on the dependent variables. Categorical regression quantifies categorical data by assigning numerical values to the categories, resulting in an optimal linear regression equation for the transformed variables. Multivariable analysis that included Pillai’s trace, Wilks’ lambda, Hotelling’s trace, and Roy’s largest root was used to test the effect of the increase in experiment time and operation repetition number on the experimental results. Then, according to the result of Mauchly’s test of sphericity (*p* < 0.05), we analyzed the corrected part of the unary analysis for studying the effect of target layout and target clicking method on picking time and dragging performance.

## Results

### Picking Time

The results of repeated measures analysis of the picking time are shown in Table 1. Multivariable analysis that included Pillai’s trace, Wilks’ lambda, Hotelling’s trace, and Roy’s largest root was used to test the effect of the increase in experiment time and operation repetition number on the experimental results. The result (*p* > 0.05) showed that the experiment time and operation repetition number did not make a difference in the five values of the target’s initial position and the two target clicking methods, which indicated that the increase in experiment time and operation repetition number did not affect the experimental results (picking time).

**TABLE 1:** The repeated measures analysis.

**Effect**	**Method**	**Value**	***F***	***p***
Picking time	Pillai’s trace	0.019	0.888	0.449
	Wilks’ lambda	0.981	0.888	0.449
	Hotelling’s trace	0.019	0.888	0.449
	Roy’s largest root	0.019	0.888	0.449
Picking time × target clicking method	Pillai’s trace	0.013	0.610	0.609
	Wilks’ lambda	0.987	0.610	0.609
	Hotelling’s trace	0.013	0.610	0.609
	Roy’s largest root	0.013	0.610	0.609
Picking time × the position of target	Pillai’s trace	0.078	0.934	0.513
	Wilks’ lambda	0.923	0.931	0.516
	Hotelling’s trace	0.081	0.927	0.520
	Roy’s largest root	0.054	1.893	0.115
Dragging time	Pillai’s trace	0.010	0.445	0.721
	Wilks’ lambda	0.990	0.445	0.721
	Hotelling’s trace	0.010	0.445	0.721
	Roy’s largest root	0.010	0.445	0.721
Dragging time × target clicking method	Pillai’s trace	0.002	0.086	0.968
	Wilks’ lambda	0.998	0.086	0.968
	Hotelling’s trace	0.002	0.086	0.968
	Roy’s largest root	0.002	0.086	0.968
Dragging time × the position of target	Pillai’s trace	0.063	0.746	0.706
	Wilks’ lambda	0.938	0.745	0.707
	Hotelling’s trace	0.065	0.744	0.708
	Roy’s largest root	0.053	1.839	0.125
Dragging numbers	Pillai’s trace	0.010	0.479	0.697
	Wilks’ lambda	0.990	0.479	0.697
	Hotelling’s trace	0.010	0.479	0.697
	Roy’s largest root	0.010	0.479	0.697
Dragging numbers × target clicking method	Pillai’s trace	0.007	0.335	0.800
	Wilks’ lambda	0.993	0.335	0.800
	Hotelling’s trace	0.007	0.335	0.800
	Roy’s largest root	0.007	0.335	0.800
Dragging numbers × the position of target	Pillai’s trace	0.069	0.821	0.628
	Wilks’ lambda	0.933	0.815	0.635
	Hotelling’s trace	0.071	0.808	0.642
	Roy’s largest root	0.041	1.418	0.231

*Tests the null hypothesis that the error covariance matrix of the orthonormalized transformed dependent variables is proportional to an identity matrix. Within subjects design: Dragging numbers.*

According to the result of Mauchly’s sphericity test (*p* < 0.05, see Table 2), we should analyze the results of the corrected tests (see Table 3). The results indicated that the position of the target (*p* = 0.083) and the target clicking method (*p* = 0.295) had no significant effect on the picking time.

**TABLE 2:** Mauchly’s sphericity test.

**Within subjects effect**	**Mauchly’s W**	**Approx. Chi-Square**	**df**	**Sig.**	**Epsilon**		
					**Greenhouse-Geisser**	**Huynh-Feldt**	**Lower-bound**
Picking time	0.663	56.994	5	0.000	0.788	0.854	0.333
Dragging time	0.770	36.226	5	0.000	0.883	0.959	0.333
Dragging numbers	0.770	36.226	5	0.000	0.883	0.959	0.333

*Tests the null hypothesis that the error covariance matrix of the orthonormalized transformed dependent variables is proportional to an identity matrix. Within subjects design: Dragging numbers.*

**TABLE 3:** Tests of within subjects.

**Variable**	**Source**	***F***	**Sig.**
Picking time	Target clicking method	1.107	0.295
	The position of target	2.111	0.083
	Target clicking method × the position of target	0.546	0.703
Dragging time	Target clicking method	6.306	0.013
	The position of target	1.480	0.211
	Target clicking method × the position of target	0.949	0.438
Dragging numbers	Target clicking method	6.306	0.013
	The position of target	1.480	0.211
	Target clicking method × the position of target	0.949	0.438

From the results of the ANOVAs, it can be seen that there was no significant difference in the picking time with different target picking methods and initial positions of the target, so the regression analysis would be meaningless for picking time.

### Dragging Time

The results of a repeated measures analysis of the dragging time are shown in Table 1. The results (*p* > 0.05) indicated that increases in experiment time and operation repetition number did not affect the experimental results (dragging time).

According to the result of Mauchly’s sphericity test (*p* < 0.05, see Table 2), we should analyze the results of corrected tests (see Table 3). The results indicated that the target clicking method (*p* = 0.013) had a significant effect on the dragging time. However, the position of the target (*p* = 0.211) and the target clicking method × the position of the target (*p* = 0.438) had no significant effect on the dragging time.

Then, the regression analysis was discussed. It can be seen from Table 4 that the overall regression model (*p* = 0.016 < 0.05) has statistical significance, i.e., overall, the regression model statistically significantly predicts the outcome variable. Table 5 shows the standardized coefficients and the test results of each coefficient in the model. The results indicated that the target clicking method (*p* = 0.001 < 0.05) had a significant effect on the dragging time and that the position of the target (*p* = 0.739) had no significant effect on the dragging time. Focusing on Table 6, the result (Importance) shows that a strong predictor is the target clicking method and that the position of the target is not a significant predictor of dragging time.

**TABLE 4:** The ANOVA of the regression model.

	**Sum of Squares**	**df**	**Mean Square**	***F***	**Sig.**
Regression^a^	10.230	3	3.410	3.562	0.016
Residual^a^	139.770	146	0.957		
Total^a^	150.000	149			
Regression^b^	8.788	4	2.197	2.256	0.066
Residual^b^	141.212	145	0.974		
Total^b^	150.000	149			

*Predictors (constant): The target clicking method, the position of target. Dependent variable: ^*a*^average dragging time; ^*b*^average dragging numbers.*

**TABLE 5:** Coefficients of regression models.

	**Standardized coefficients**		**df**	***F***	**Sig.**
	**Beta**	**Bootstrap (1000) estimate of std. error**			
The target clicking method^a^	−0.255	0.092	2	7.753	0.001
The position of target^a^	−0.055	0.165	1	0.112	0.739
The target clicking method^b^	−0.233	0.083	2	7.886	0.001
The position of target^b^	0.067	0.153	2	0.189	0.828

*Dependent variable: ^*a*^average dragging time; ^*b*^average dragging numbers.*

**TABLE 6:** Correlations and tolerance of the dragging time.

	**Correlations**			**Importance**	**Tolerance**	
	**Zero-order**	**Partial**	**Part**		**After transformation**	**Before transformation**
The target clicking method^a^	−0.255	−0.256	−0.255	0.955	1.000	1.000
The position of target^a^	−0.055	−0.057	−0.055	0.045	1.000	1.000
The target clicking method^b^	−0.233	−0.233	−0.233	0.924	1.000	1.000
The position of target^b^	0.067	0.069	0.067	0.076	1.000	1.000

*Dependent variable: ^*a*^average dragging time; ^*b*^average dragging numbers.*

### Dragging Numbers

The results of a repeated measures analysis of the dragging numbers are shown in Table 1. The result (*p* > 0.05) indicated that the increase in experiment time and operation repetition number did not affect the experimental results (dragging numbers).

According to the result of Mauchly’s sphericity test (*p* < 0.05, see Table 2), we should analyze the results of corrected tests (see Table 3). The results indicated that the target clicking method (*p* = 0.013) had a significant effect on the dragging numbers. However, the position of the target (*p* = 0.211) and the target clicking method × the position of the target (*p* = 0.438) had no significant effect on the dragging numbers.

The target clicking method had a significant effect on the dragging numbers; therefore, regression analysis was carried out. Table 4 shows that the overall regression model (*p* = 0.066) has no statistical significance. However, we can see the effect of each of the predictors on the dragging numbers in Table 5. The result shows that the target clicking method (*p* = 0.001 < 0.05) had a significant effect on the dragging numbers and that the position of the target (*p* = 0.828) had no significant effect on the dragging numbers. Table 6 shows that a strong predictor is the target clicking method and that the position of the target is not a significant predictor of dragging numbers.

### Target Picking Method

**FIGURE 3:**
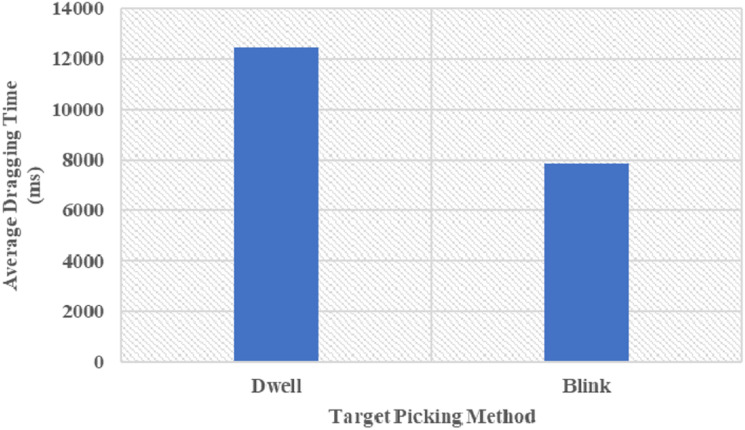
Comparison of the two target picking methods.

Through the analyses of the results, it can be concluded that the target picking method has significant effects on the dragging time and the dragging numbers. A comparison of the two target picking methods is shown in Figure 3. When the target picking method is *dwell*, the manipulation for the interface is better, the time of the complete dragging command is shorter, and the error rate is lower.

## Discussion

The results show that the target layout has no significant effect on the picking time and drag performance (dragging time and dragging numbers), which is inconsistent with our previous assumptions, so Hypothesis 2 cannot be accepted at the significance level of 0.05, which may be related to the set experimental conditions (such as light level and screen resolution). However, the target clicking method (blinking and dwell) has a significant effect on picking dragging time and the dragging number. The experimental results agree with Hypothesis 3. The target clicking method and target layout have no significant interaction impact on picking time and drag performance, which is consistent with Hypothesis 4.

According to the results of CATREG, the overall regression model has statistical significance. The relationships between the target clicking method and the dragging time and numbers are statistically significant. However, the position of the target had no significant effect on the dragging time and numbers. The effect of the target clicking method on dragging performance is stronger than the effect of the initial position of the target.

## Conclusion

The study considers the influence of experiment time and operation repetition number on the experiment performance, and evaluates the impact of target layout and clicking method on picking time and drag performance from the perspective of efficiency and effectiveness. The experimental results show that the target layout has no significant effect on the picking time and drag performance (dragging time and drag numbers). However, the target click method, i.e., blink and dwell, has a significant impact on the dragging time and numbers, and compared to using blink, using dwell to click can identify the target better, with shorter time and higher accuracy. The findings are anticipated to provide helpful implications for future eye control technique design and HCI interface design.

## Data Availability Statement

The datasets analyzed in this article are not publicly available because of privacy policies. Requests to access the datasets should be directed to JN, niujw@ustb.edu.cn.

## Ethics Statement

The studies involving human participants were reviewed and approved by the University of Science and Technology Beijing, Beijing, China. The patients/participants provided their written informed consent to participate in this study.

## Author Contributions

LW and DW contributed to validation, formal analysis, writing—original draft preparation, and writing—review and editing. YZ contributed to conceptualization, methodology, project administration, and funding acquisition. HL contributed to methodology, software, and visualization. JS contributed to methodology and formal analysis. YZ contributed to methodology and validation. CZ contributed to conceptualization, methodology, and formal analysis. JN contributed to conceptualization, methodology, formal analysis, writing—review and editing, and funding acquisition. All authors contributed to the article and approved the submitted version.

## Conflict of Interest

The authors declare that the research was conducted in the absence of any commercial or financial relationships that could be construed as a potential conflict of interest.
